# Structural mechanism of VWF D’D3 dimer formation

**DOI:** 10.1038/s41421-022-00378-2

**Published:** 2022-02-15

**Authors:** Zimei Shu, Jianwei Zeng, Li Xia, Haiyan Cai, Aiwu Zhou

**Affiliations:** 1 Department of Pathophysiology, Key Laboratory of Cell Differentiation and Apoptosis of Chinese Ministry of Education, Shanghai Jiao Tong University School of Medicine, Shanghai, China; 2 State Key Laboratory of Membrane Biology, Beijing Advanced Innovation Center for Structural Biology, School of Life Sciences, Tsinghua University, Beijing, China; 3 Present Address: Department of Biochemistry and Molecular Biophysics, Washington University in St. Louis, School of Medicine, St. Louis, MO USA

**Keywords:** Cryoelectron microscopy, Protein folding

Dear Editor,

Von Willebrand factor (VWF) assembly begins in the endoplasmic reticulum of endothelial cells and megakaryocytes where VWF is synthesized as a precursor with multiple domains (Fig. 1a). The polypeptide including the propeptide and mature VWF chain (proVWF) subsequently forms “tail-to-tail” homodimers through their C-terminal cystine knot (CK) domains. These proVWF dimers are then transported to the Golgi where they assemble into large multimers ‘head-to-head’ through interchain disulfide bonds between D3 domains of two proVWF dimers^1–3^. The Sadler group firstly identified a peptide containing the Cys^1142^–Cys^1142′^ disulfide bond from plasma multimeric VWF^4^. Subsequently, through differential alkylation, proteolytic digestion, and mass spectrometry, they identified two free cysteines Cys^1099^ and Cys^1142^ from D′D3 monomer and proposed their involvement in D′D3 dimer formation^5^. However, the crystal structure of a monomeric D′D3 mutant (C1099A/C1142A) reported by Dong and Springer revealed that residue Cys^1099^ is largely buried inside of the D′D3 domain and it has to undergo a dramatic conformational change to allow forming a disulfide bond with the same cysteine from the other D′D3 molecule^6,7^. Furthermore, the structures of dimeric D3 domains of Mucin 2 (MUC2), a homologous multimeric protein of VWF, showed Cys^1142^–Cys^1142′^ and Cys^1097^–Cys^1097′^ disulfide linkages (using VWF residue numbering)^8^. Cys^1091^ formed an intramolecular disulfide bond with Cys^1099^ in MUC2 instead of the Cys^1091^–Cys^1097^ seen in the crystal structure of the VWF D′D3 mutant. Therefore, the disulfide linkages and the cysteines involved in the VWF D′D3 dimer interface remain to be confirmed with the discrepancy mainly on Cys^1097^ and Cys^1099^ (Fig. 1a). Here we identified the disulfide linkages from the D′D3 dimer interface through chemical digestion and mass spectrometry and solved the cryo-electron microscopy (cryo-EM) structure of VWF tubules with each repeating unit containing one D′D3 dimer and one D1D2 dimer.

**Fig. 1:**
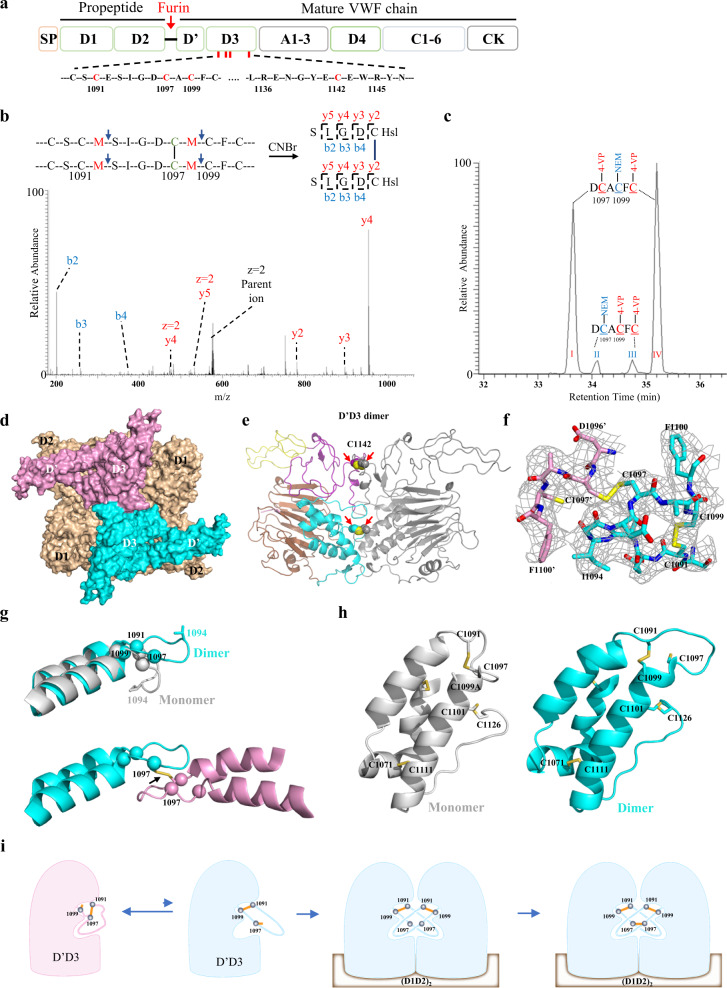
Structural mechanism of D′D3 dimer formation. **a** The domain arrangements of full-length VWF. **b** Identification of disulfide-linked peptides from the D′D3 dimer interface. The D′D3 dimers were cleaved by CNBr and analyzed by mass spectrometry with Cys^1097^–Cys^1097′^ peptides confirmed by the MS/MS spectrum. **c** Identification of free cysteines in monomeric D′D3. D′D3 monomers were sequentially modified by NEM and 4-VP, then treated with protease Asp-N and analyzed by LC-MS. **d** Cryo-EM structure of the repeating unit containing one VWF D′D3 dimer (cyan and pink) and one D1D2 dimer (wheat), which mirrors the repeating unit of MUC2. Other intermolecular interfaces in the complex are shown in Supplementary Fig. S8. **e** The dimer shown in the cartoon with the VWD3 module of D3 (left chain) colored in brown, C8-3 in cyan, TIL3 in purple, and E3 in yellow and the other D3 chain in gray. **f** The electron densities covering the key disulfide bonds Cys^1097^–Cys^1097’^ and Cys^1091^–Cys^1099^ near the D′D3 dimeric interface are shown as gray mesh contoured at 1.5 rmsd. **g** The overlaid structures of D′D3 monomer (gray) and dimer (cyan) showing the loop linking residues 1091 and 1099 flips up and stacks with the same loop from the other molecule in the dimer (Supplementary Fig. S5). **h** Relative positions of cysteines Cys^1091^, Cys^1097^, and Cys^1099^ in D′D3 monomer and dimer. **i** A diagram showing D′D3 monomer equilibrating in configurations with either Cys^1097^ or Cys^1099^ free. Only the main form with free Cys^1099^ was identified in the previous study possibly due to the lower sensitivity of the equipment or the lower amount of protein used. In the presence of VWF D1D2 domains at mild acidic pH, only D′D3 monomers with Cys^1091^–Cys^1099^ bond could readily bind each other complimentarily and allow intermolecular Cys^1097^–Cys^1097’^ disulfide bond formation.

As the disulfide linkage of Cys^1099^ or Cys^1097^ from the VWF D′D3 dimer interface has never been identified and no suitable enzymic cleavage sites near the dimeric interface could be utilized to distinguish the cysteines of our interest, namely Cys^1091^, Cys^1097^, and Cys^1099^, here we designed a series of D′D3 mutants with residues flanking Cys^1097^, Cys^1099^, or Cys^1142^ mutated to methionines (Fig. 1b), and identified the intermolecular disulfide linkages directly by CNBr cleavage and mass spectrometry. For the D′D3 dimer variant with Glu^1092^ and Ala^1098^ flanking Cys^1097^ mutated to methionines (D′D3-E1092M-A1098M), a doubly charged parent ion with *m/z* = 576.223 corresponding to the predicted Cys^1097^–Cys^1097′^ disulfide-linked peptide (^1093^SIGDC (Hsl)^1098^)_2_ where M was converted to homoserine lactone (Hsl) could be readily identified. The identity of this peptide was further confirmed by the MS/MS spectrum of this ion with all the expected ions of peptide fragments detected such as y4 (*m/z* = 951.321, z = 1) and b2 (*m/z* = 201.124, z = 1) (Fig. 1b). Similarly, a doubly charged ion with *m/z* = 796.270 was identified from the dimer of the D′D3 variant (D′D3-R1136M-E1143M), which corresponded to the Cys^1142^–Cys^1142′^ disulfide-linked peptide (^1137^ENGYEC(Hsl)^1143^)_2_ (Supplementary Fig. S1a). Notably, the disulfide-linked peptides where one or two methionines were converted to homoserines could also be readily identified (Supplementary Fig. S1b, c). Therefore, these results unequivocally demonstrated the presence of Cys^1097^–Cys^1097′^ and Cys^1142^–Cys^1142′^ linkages in the VWF D′D3 dimers.

We then tried to verify the free cysteines in the D′D3 monomer using the same differential alkylation procedure reported previously where D′D3 monomers were treated with N-ethylmaleimide (NEM) to block free thiols and the intramolecular disulfide bonds were reduced with dithiothreitol (DTT) and modified by 4-vinylpyridine (4-VP)^5^. The peptides (^1137^ENGYEC(NEM)EWR^1145^) with Cys^1142^ modified by NEM could be readily detected from the tryptic digestion (data not shown). Interestingly, four peptide peaks were identified from the Asp-N digestion by liquid chromatography with identical doubly charged ions with *m/z* = 498.675, which corresponded to peptide ^1096^DCACFC^1101^ modified by one NEM and two 4-VP molecules (Fig. 1c). The peptides in the two major peaks (I and IV) were confirmed by the MS/MS spectrum to be ^1096^DC(4-VP)AC(NEM)FC(4-VP) ^1101^ with two peaks representing two diastereomers from the NEM derivative. This is consistent with previous findings that Cys^1099^ in D′D3 monomer could be modified by NEM^5^. The peptides in the two minor peaks (II, III) were identified to be ^1096^DC(NEM)AC(4-VP)FC(4-VP)^1101^, corresponding to those with Cys^1097^ modified by NEM (Fig. 1c). These results indicate that Cys^1099^ is the free cysteine in most D′D3 monomers, but Cys^1097^ is free in a small amount of D′D3 monomers. Thus, it is highly plausible that there is an equilibrium of Cys^1099^ and Cys^1097^ forming an alternative disulfide bond with the same cysteine, most likely Cys^1091^, which may be consistent with the disulfide exchange mechanism proposed by Dong and Springer^6,7^. Notably, the replacement of either Cys^1097^ or Cys^1142^ alone would not prevent D′D3 dimerization, consistent with a similar mutagenesis study of MUC2^8^ (Supplementary Fig. S2).

In order to gain further structural information of the D′D3 dimer interface, we purified D′D3 dimers complexed with D1D2 derived from the expression medium of HEK293 cells transfected with expression plasmid of D1D2D′D3-wt or D1D2D′D3-R1136M-E1143M. The tubule-like oligomers of these complexes were analyzed by single-particle cryo-EM (Supplementary Figs. S3, S4). This yielded an electron density map covering two repeating units for D′D3-R1136M-E1143M dimer complexed with D1D2 at 3.3 Å resolution and an electron density map covering one repeating unit for D′D3-wt dimer complexed with D1D2 at 3.4 Å resolution respectively (Supplementary Table S1). Each repeating unit contains a D′D3 dimer and a D1D2 dimer. As the overall configuration of the refined D′D3-wt complex is essentially the same as that of the D′D3-R1136M-E1143M mutant, the structure of the mutant was selected for presentation due to its slightly better resolution. The shape of the repeating unit largely resembles that of MUC2 where a D′D3 dimer is docked in the central hole of the donut-shaped D1D2 dimer^8^ (Fig. 1d, e). Since the crystal structure of a monomeric D′D3 variant has been solved previously^6^, this allowed us to build the D′D3 dimer structure with confidence (Fig. 1e; Supplementary Table S1). The electron density near the center covering the D′D3 dimer is unambiguous and a clear density covering the disulfide bonds near the dimeric interface can be observed (Fig. 1f).

The structure (Fig. 1e) showed that D′D3 retained the overall conformation seen in the crystal structure of D′D3 monomer (rmsd of 1.7 Å) but with local conformational rearrangements near the dimeric interface (Fig. 1g, h and Supplementary Figs. S5, S6). The connecting loop linking two helices in the D′D3 monomer restrained by the Cys^1091^–Cys^1097^ disulfide is anchored in a hydrophobic pocket formed by Phe^1100^, Met^1055^, and Val^1056^ through Ile^1094^, but in the dimer, it is more flexible with the intramolecular Cys^1091^–Cys^1099^ bond and flips over to dock in the same hydrophobic surface pocket of the other D′D3 molecule in a domain swapping-like fashion (Supplementary Fig. S6). This brings two Cys^1097^ residues in proximity and the subsequent formation of an intermolecular disulfide bond. As Cys^1142^ in a double-stranded hairpin was shielded from solvent exposure in the D′D3 monomer structure^6^, but rotated and shifted ~6 Å to form an intermolecular disulfide bond with its counterpart in the other D′D3 molecule in the dimer (Supplementary Figs. S5, S6), it seems that VWF has adopted an allosteric mechanism for the intermolecular disulfide formation with the free thiols in the monomers protected from surface exposure avoiding unwanted oxidation during the biosynthesis.

Overall, our studies here indicate that D′D3 monomer equilibriums between two configurations where Cys^1091^ forms an intramolecular disulfide bond with either Cys^1097^ or Cys^1099^ (Fig. 1i). However, only the minor D′D3 confirmation containing a longer connecting loop with the Cys^1091^–Cys^1099^ bond and free Cys^1097^ could allow domain swapping-like complementary binding of the two loops in the dimer interface and the formation of intermolecular Cys^1097^–Cys^1097’^ disulfide bond in the presence of VWF domain D1D2s (Fig. 1i). As there are very limited noncovalent interactions in the D′D3 dimeric interface (Supplementary Fig. S6), D1D2 is indispensable for its dimerization by aligning two D3 monomers in optimal positions for their intermolecular disulfide formation (Fig. 1d, i). Although it remains unclear how the disulfides in the D′D3 dimer interface would reshuffle or form at ~pH 5.8 in Golgi and various mechanisms have been proposed^7,9^, our cryo-EM structure of a D′D3 dimer complexed with D1D2s shows that the two Cys^1097^ are largely buried in the center of the dimeric interface and there is limited space to allow the participation of any bulky redox-regulating enzymes (Supplementary Figs. S7, S8). Therefore, it is plausible that only small redox molecules are directly involved in this process, which would be consistent with the previously observed formation of VWF oligomers in vitro^10^.

## Supplementary information

Supplementary Figures and Methods
