# Supplementary material for: Structural mechanism of VWF D’D3 dimer formation
Source: Cell Discov. 2022 Feb 15;8:14. doi: 10.1038/s41421-022-00378-2 (PMC8847404; doi:10.1038/s41421-022-00378-2)
Supplement: Supplementary file 1 — Supplementary Figures and Methods [file 41421_2022_378_MOESM1_ESM.pdf]

a

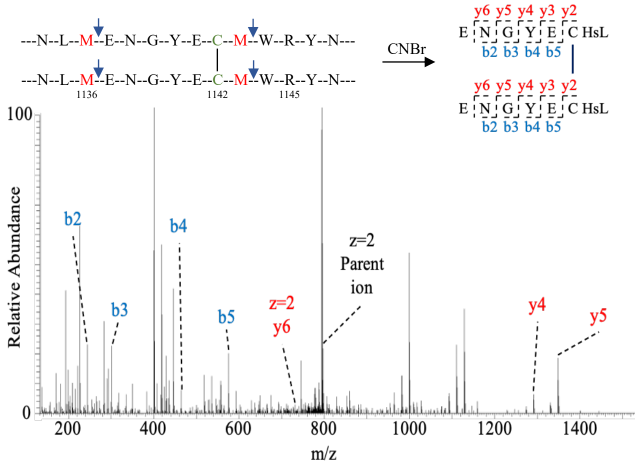

b

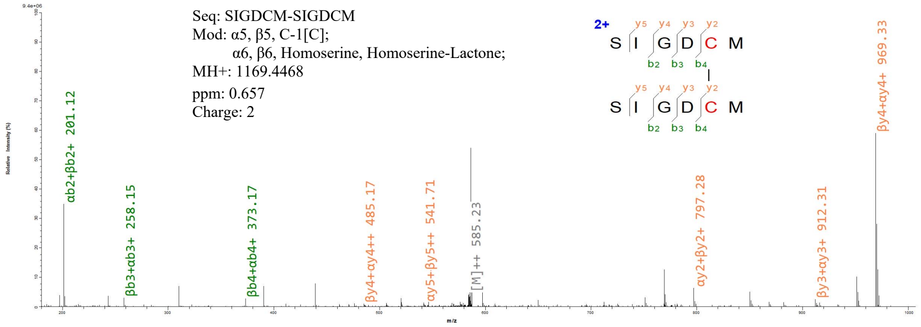

c

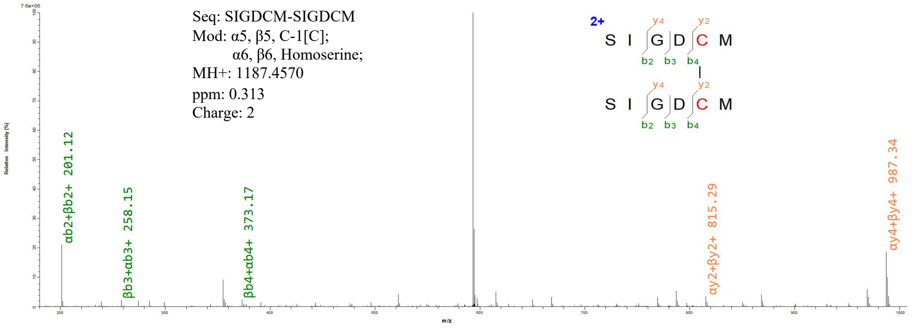

Supplementary Figure S1: Detailed MS/MS spectrum of the peptides containing Cys[1142]-Cys[1142'] disulfide linkages with both methionines converted to homoserine lactone **(a)**. The MS/MS spectrum of Cys[1097]-Cys[1097'] disulfide linkages where one methionine was converted to homoserine and the other converted to homoserine lactone (**b**) or both methionines were converted to homoserine (**c**).

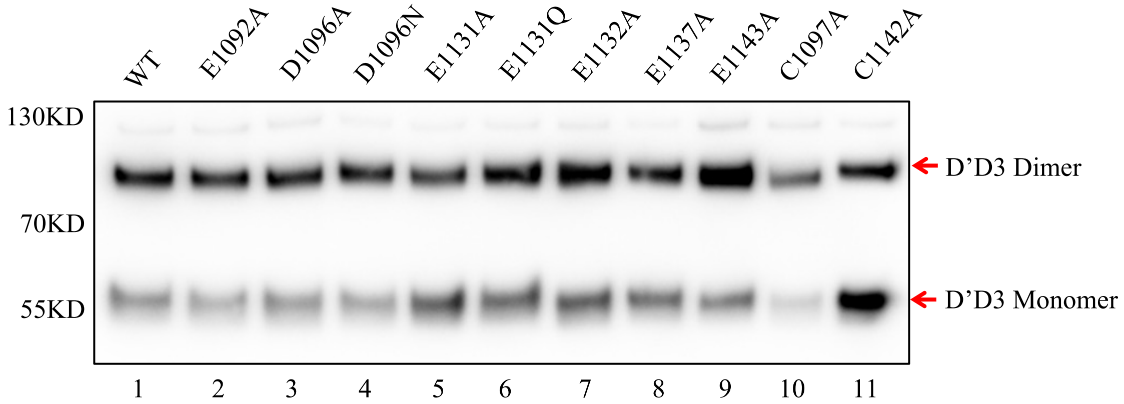

Supplementary Figure S2. Impact of residue substitution in the dimeric interface on D’D3 dimer formation. Various single point mutation was introduced into the D1D2D’D3 fragment with a His-tag at the C-terminus (D1D2D’D3-HisTag) and the expression medium of the mutants from HEK293 cells were analyzed by non-reducing SDS-PAGE and western blot using anti-His-tag antibody. D’D3 monomer and dimer could be detected from the medium of the wild type with D1D2 removed by the intrinsic furin during the secretion process. Both C1097A and C1142A retained the ability to form dimers, which is consistent with previous observation with Mucin 2, a homologous protein of VWF.

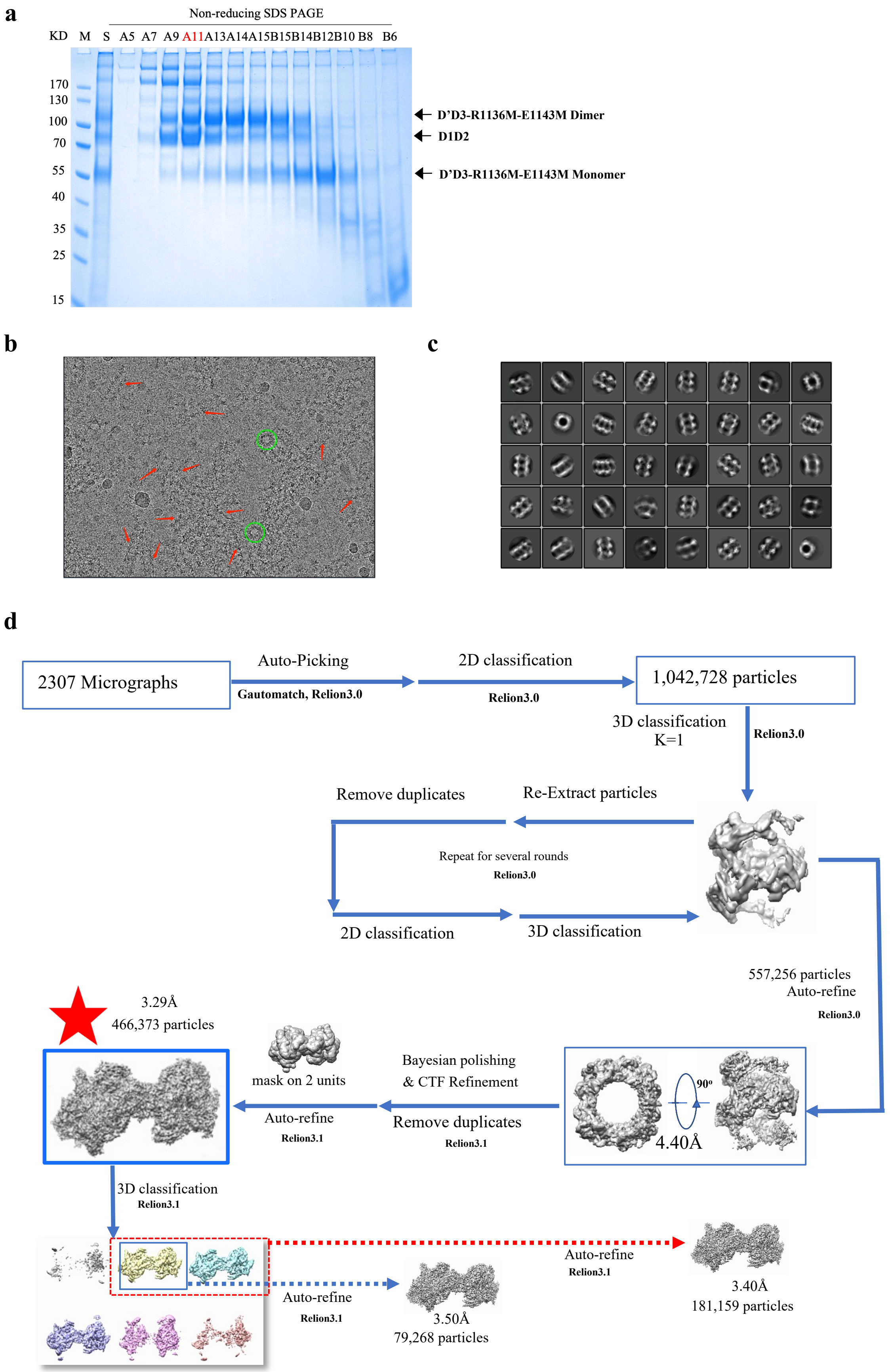
a
Non-reducing SDS PAGE
KD M S A5 A7 A9 A11 A13 A14 A15 B15 B14 B12 B10 B8 B6
170
130
100
70
55
40
35
25
15
D'D3-R1136M-E1143M Dimer
D1D2
D'D3-R1136M-E1143M Monomer
b
c
d
2307 Micrographs
Auto-Picking
Gautomatch, Relion3.0
2D classification
Relion3.0
1,042,728 particles
3D classification
K=1
Relion3.0
Remove duplicates
Re-Extract particles
Repeat for several rounds
Relion3.0
2D classification
3D classification
557,256 particles
Auto-refine
Relion3.0
3.29Å
466,373 particles
mask on 2 units
Bayesian polishing
& CTF Refinement
Auto-refine
Relion3.1
Remove duplicates
Relion3.1
90°
4.40Å
3D classification
Relion3.1
Auto-refine
Relion3.1
3.40Å
181,159 particles
Auto-refine
Relion3.1
3.50Å
79,268 particles

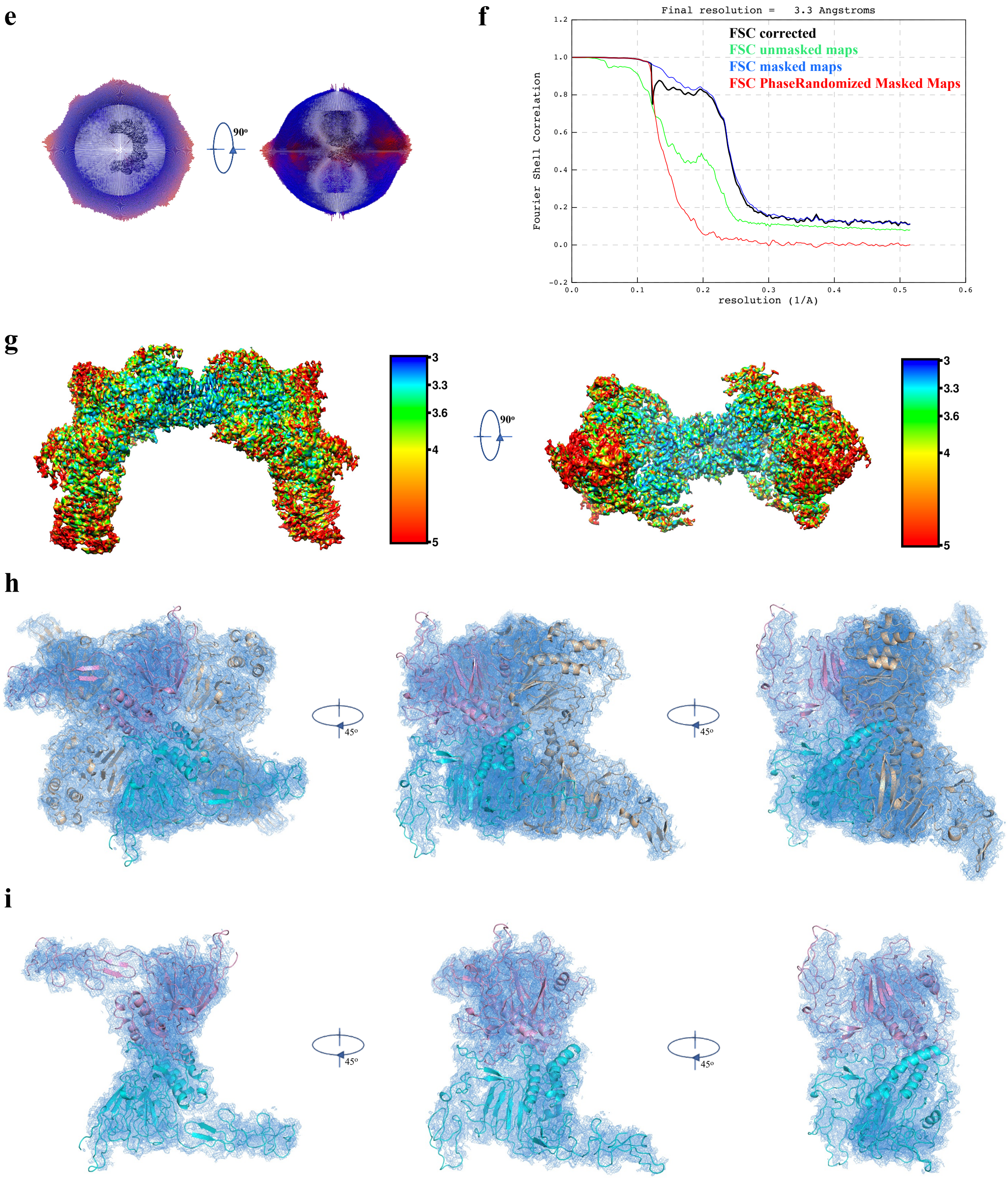

Supplementary Figure S3: Cryo-EM analysis of the VWF D'D3-R1136M/E1143M dimer complexed with D1D2 dimer. **a** Purification of D'D3 dimer complexed with D1D2. The expression medium from HEK293 transfected with D1D2D'D3-R1136M-E1143M-His or D1D2D'D3-wt-His expression plasmid was loaded onto a Ni-chelating column. The bound protein was eluted with imidazole and then loaded onto a Superdex 200 gel filtration column with all the fractions analyzed by non-reducing SDS-PAGE. The fraction containing both D'D3 dimer and D1D2 (A11) was analyzed by Cryo-EM. **b** A representative Cryo-EM micrograph on 300kV Titan Krios. Top view particles were picked with green cycles, diameter=350Å; side view particles or filament were pointed out with red arrows. **c** Representative 2D class averages. **d** Flowchart for the Cryo-EM data processing of the VWF D'D3-R1136M/E1143M dimer complexed with a D1D2 dimer. The density map of 2-unit at 3.29Å resolution is indicated by a red star. **e** Angular distribution of Cryo-EM particles used for final structural refinement. **f** The gold-standard Fourier shell correlation (FSC) curves for the 3D reconstruction. **g** Local resolution map for the 3D EM reconstruction of two repeating unit. **h** The overall fitting of D'D3-D1D2 hetero-tetramer complex model in the Cryo-EM electron density (blue mesh) contoured at 1.0 rmsd. **i** The electron density covering the D'D3-dimer is carved out and contoured at 1.0 rmsd.

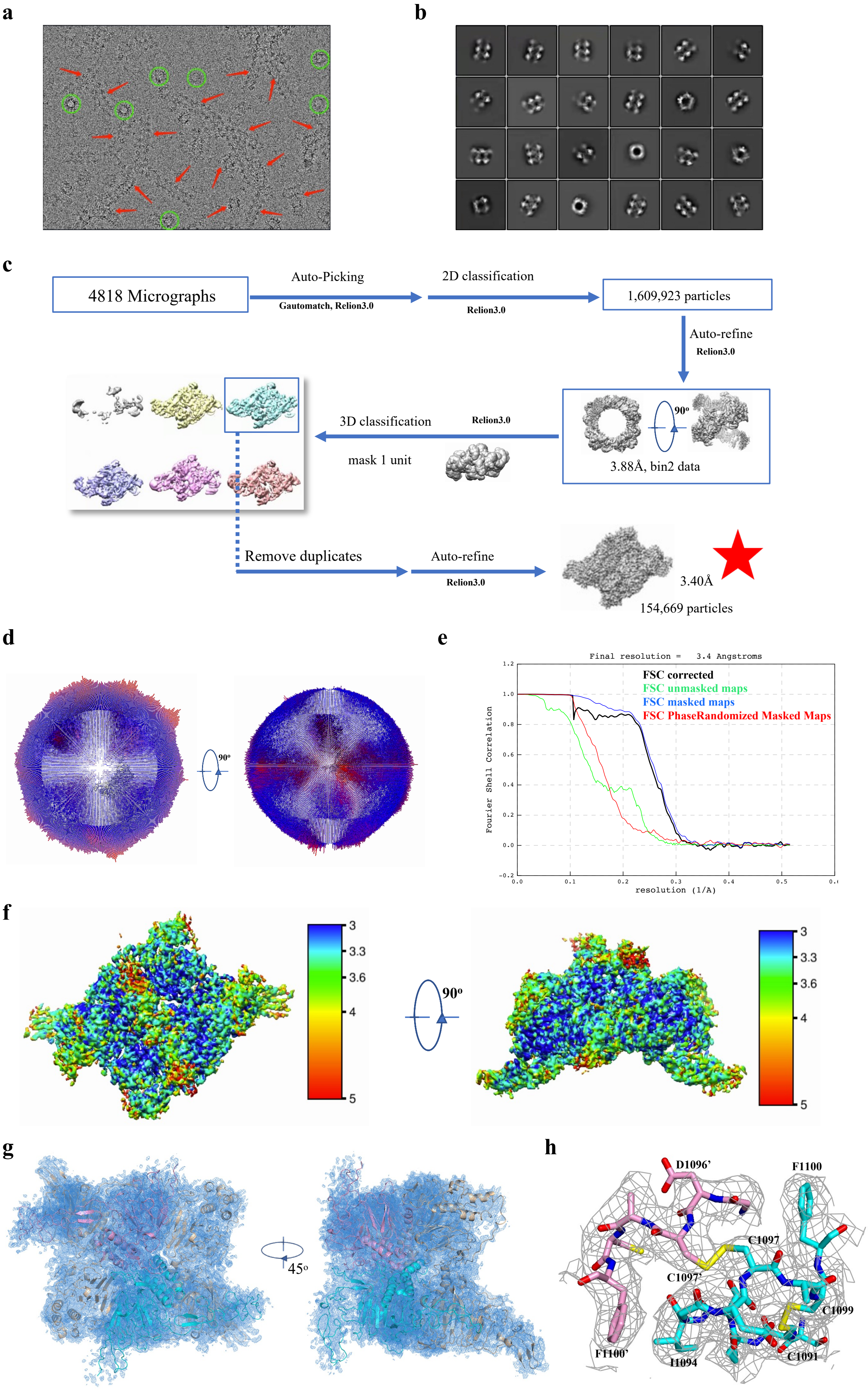
a
b
c
4818 Micrographs
Auto-Picking
Gautomatch, Relion3.0
2D classification
Relion3.0
1,609,923 particles
Auto-refine
Relion3.0
90º
3.88Å, bin2 data
3D classification
Relion3.0
mask 1 unit
Remove duplicates
Auto-refine
Relion3.0
3.40Å
154,669 particles
d
90º
e
Final resolution = 3.4 Angstroms
FSC corrected
FSC unmasked maps
FSC masked maps
FSC PhaseRandomized Masked Maps
Fourier Shell Correlation
resolution (1/A)
f
3
3.3
3.6
4
5
90º
g
45º
h
D1096'
F1100
C1097
C1097'
C1099
F1100'
I1094
C1091

Supplementary Figure S4: Cryo-EM analysis of the VWF D'D3-wt dimer complexed with D1D2. **a** A representative Cryo-EM micrograph on 300kV Titan Krios. Top view particles were picked with green cycles, diameter=350Å; side view particles or filament were pointed out with red arrows. **b** Representative 2D class averages. **c** Flowchart for the Cryo-EM data processing of the VWF D'D3-wt dimer complexed with D1D2. The 1-unit map at 3.4Å resolution is indicated by a red star. **d** Angular distribution of Cryo-EM particles used for final structural refinement. **e** The gold-standard Fourier shell correlation (FSC) curves for the 3D reconstruction. **f** Local resolution map for the 3D EM reconstruction of the one repeating unit. **g** The overall fitting of D'D3-D1D2 hetero-tetramer complex model in the Cryo-EM electron density (blue mesh). This structure is essentially identical to that of D'D3-R1136M/E1143M dimer complexed with D1D2. **h** The electron densities covering the key disulfide bonds Cys^{1097}-$Cys^{1097'}$ and Cys^{1091}-Cys^{1099} (in sticks) near the wt-D'D3 dimeric interface are shown as grey mesh contoured at 1.5 rmsd. Nitrogen atoms are colored in blue, oxygen atoms in red and sulfur atoms in yellow. These disulfide linkages are same as those of D'D3-R1136M/E1143M dimer (Fig. 1f).

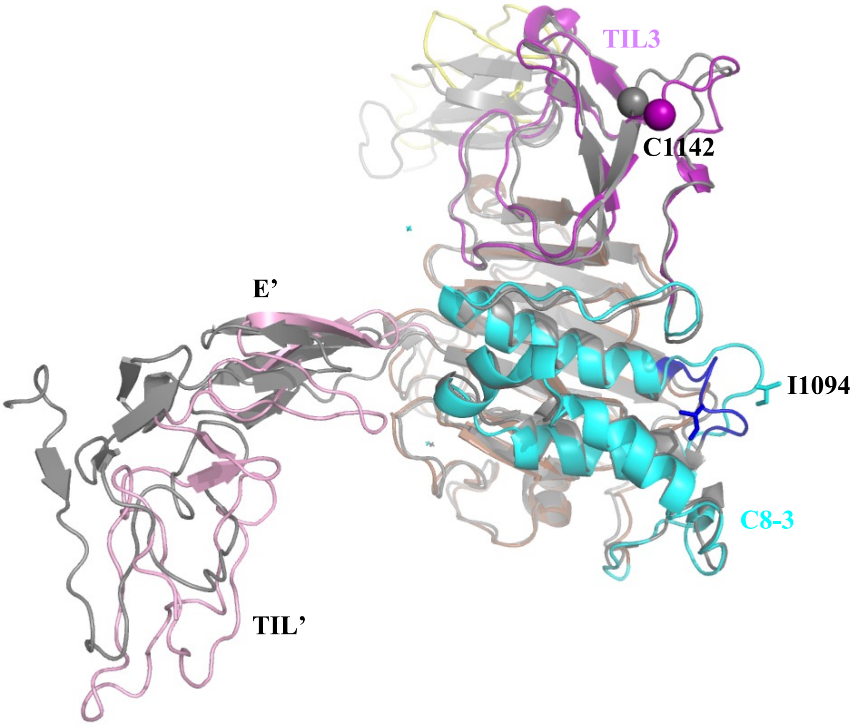

Supplementary Figure S5: Conformational changes of D’D3 during dimerization. The connecting loop consisting residues 1091-1099 (colored blue in the D’D3 monomer) flipped over during dimer formation. Cys1142 shifted about 6Å to form a disulfide bond with Cys1142 from the opposite D’D3 molecule. The monomeric D’D3 is colored in grey. The modules of TIL’, E’, VWD3, C8-3, TIL3, E3 from the dimeric D’D3 were colored in pink, brown, cyan, purple and yellow respectively. The TIL’ module of the D’ domain rotated and twisted when stacked on D2 during the quadruple complex formation.

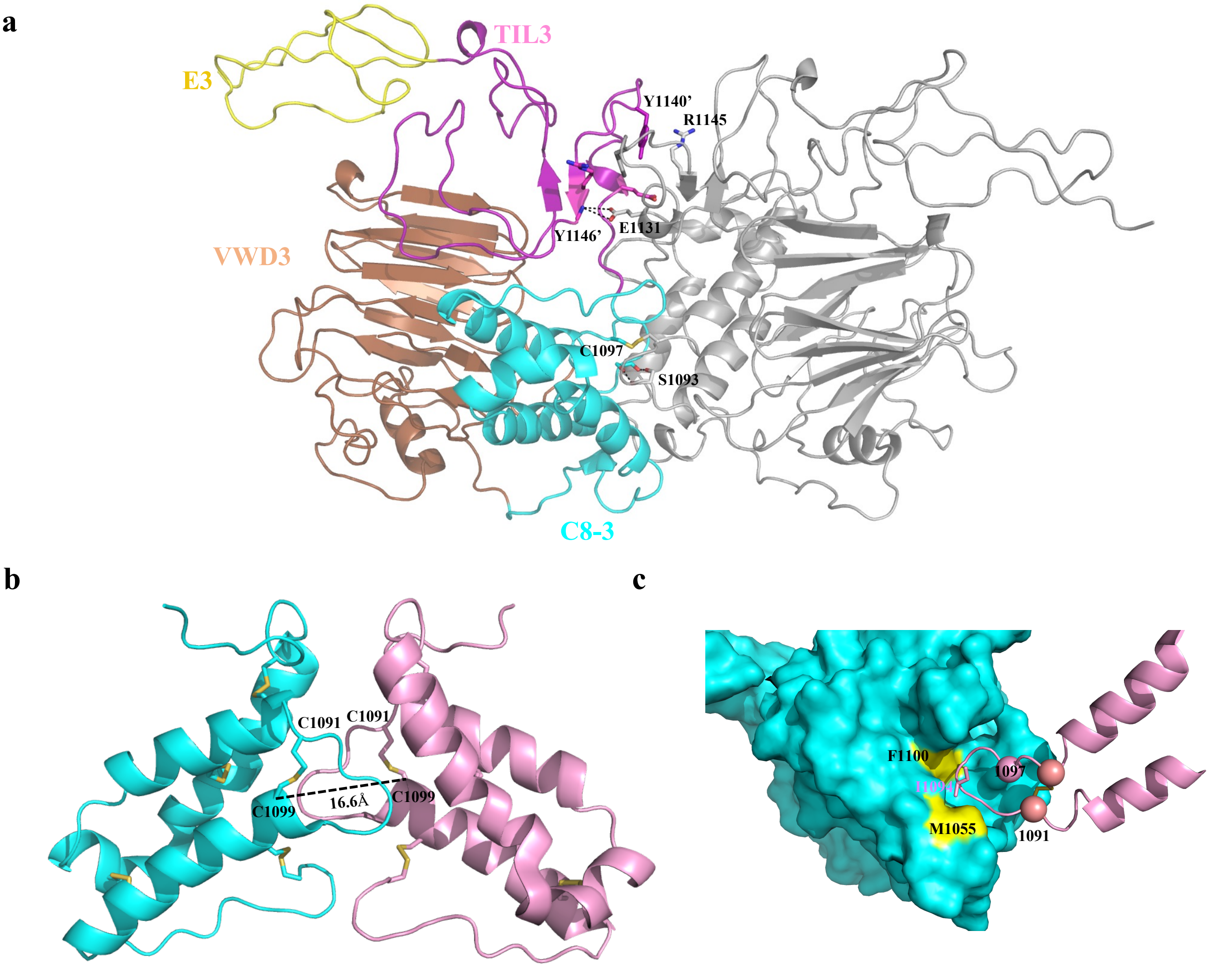

Supplementary Figure S6: The dimeric interface of D’D3. a Apart from the two disulfide bonds and the hydrophobic interactions from the Cys^{1097} containing loops, there are only several intermolecular interactions, such as sidechain to mainchain hydrogen bonds involving sidechain of Glu^{1131} with the mainchain nitrogen atom of Tyr$^{1146’}$, and sidechain of Ser^{1093} with the mainchain oxygen atom of Ser$^{1093’}$, and intermolecular cation-pi interactions formed by Arg^{1145} and Tyr$^{1140’}$. These limited noncovalent interactions between D3 dimer interface may explain why D1D2 is required for its dimerization. **b** Cys^{1099} forms an intramolecular disulfide bond with Cys^{1091} in the D’D3 dimer (shown in sticks) and the distance between the two Cys^{1099} is ~16.6Å (shown in dotted line). **c** In the D’D3 dimer, the connecting loop (Cys^{1091}-Cys^{1099}) of one molecule is docked in a hydrophobic surface pocket in the other molecule formed by Phe^{1100}, Met^{1055} and Val^{1056} (colored in yellow). This hydrophobic pocket in monomeric D’D3 is occupied by its own connecting loop as seen in the crystal structure of monomeric D’D3. Therefore, loop flipping during dimerization (Fig. 1g and 1h) allows domain-swap like complimentary binding of D’D3 with subsequent intermolecular disulfide formation.

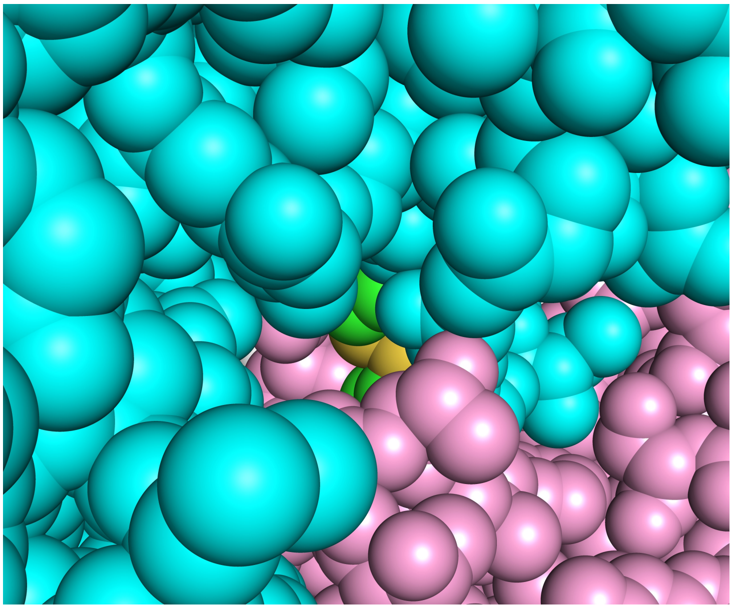

Supplementary Figure S7: The Cys^{1097}-Cys$^{1097'}$ disulfide bond is located in the center of the dimer interface with limited accessibility for bulky redox enzymes. The sulfur atom of Cys^{1097} is shown in yellow spheres with carbon atoms in green.

a

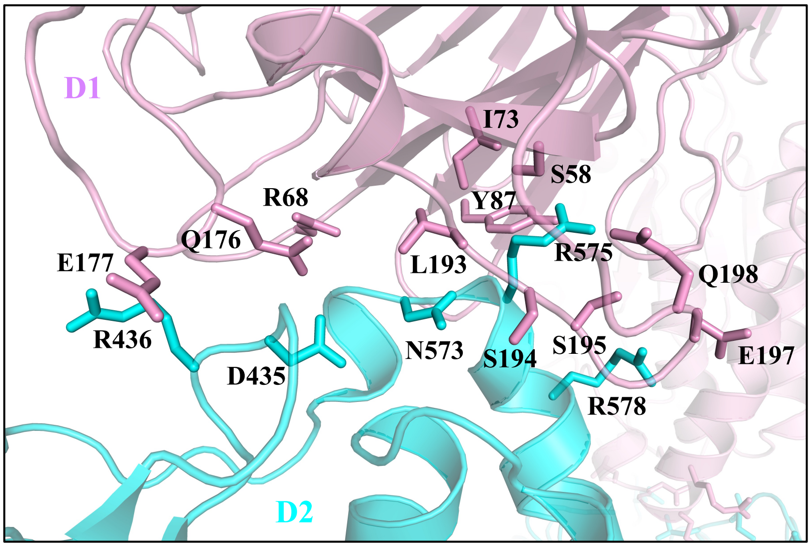

b

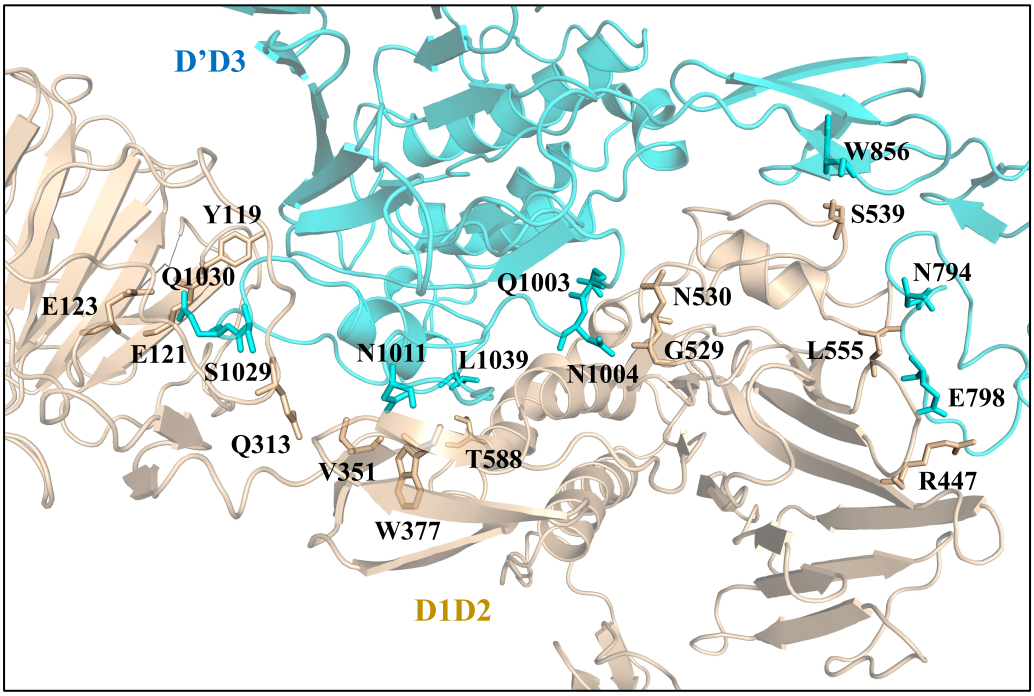

Supplementary Figure S8 : The intermolecular interfaces in the quadruple complex containing a D'D3-R1136M-E1143M dimer and a D1D2 dimer. **a** The D1:D2 binding interface involves several polar interactions from R68, N176, E197, D435, R436, N573, R575, R578. Notably, R575 in D2 is inserted in a surface pocket of D1 involving residues S58, I73, Y87, L193, S195 and Q198. The structures are shown in cartoon with D1 colored in pink and D2 in cyan. **b** The interface between D1D2 and D'D3 involves about 9 hydrogen bonds from residues such as N794, Q1030, N1011, N530, E121, E123 etc, and salt bridges from E798 and R447. All the residues of interest are shown in sticks. D1D2 shown in cartoon is colored wheat and D'D3 colored in cyan.

dataset Name(Sample Name)	D'D3-R1136M-E1143M dimer complexed with D1D2	D'D3-wt dimer complexed with D1D2
Grid	Quantifoil Cu R1.2/1.3 + 2 nm Carbon, 300 mesh	Quantifoil Au R1.2/1.3, 300 mesh
Data collection and processing		
Voltage (kV)	300	
Electron exposure (e^{-}/Å^2)	50	
Defocus range (μm)	-1.0~-2.0	
Pixel size (Å)	0.97	
Symmetry imposed	C1	
Final dataset (# of particles)	466,373	154,669
Map resolution (Å) $FSC_{0.143}$	3.3	3.4
Map-sharpening B factor (Å^2)	-24.3	-109
Model composition		
Nonhydrogen atoms	70604	35209
Protein residues	4756	2372
Ligands	36	16
Validation		
R.m.s. deviations		
Bond lengths (Å)	0.007	0.013
Bond angles (°)	1.387	1.815
Ramachandran plot (%)		
Favored	88.63	88.79
Allowed	11.37	11.21
Outliers	0.00	0.00
	PDB 7WN6	PDB 7WN4

Supplementary Table S1. Cryo-EM data collection, refinement and validation for the D'D3-wt dimer or D'D3-R1136M-E1143M dimer complexed with D1D2 dimer.

Methods

Protein preparation

Recombinant VWF D'D3 variants were prepared from HEK293 cells[1] using expression plasmid coding the residues 1-1270aa (D1D2D'D3) or 764-1270aa (D'D3) of human VWF with a His-tag at the C-terminus. The DNA sequence was cloned into expression vector pCEP4 with mutations generated by KOD-Plus-Mutagenesis Kit (Code No.SMK-101, TOYOKO). The D'D3 monomers and dimers were purified by Ni-Chelating and size-exclusion chromatography with the final elution in 10mM Tris-HCl pH7.4, 100mM NaCl. Recombinant human VWF D1D2 (residues 1-763aa) was also prepared from HEK293 cells using expression vector pCEP4 with a His-tag at the C-terminus. The D1D2 monomers were purified by Ni-Chelating and anion exchange column, and stored in 10mM Tris-HCl pH7.4, 100mM NaCl.

CNBr digestion, thiol modification and mass spectrometry

The purified D'D3 dimers with designated methionine mutations, which were selected based on the crystal structure of D'D3 monomer (PDB 6N29), were mixed with CNBr (Sigma) at approximate 1:50 molar ratio in 0.1M HCl at room temperature for 16h in dark before mass spectrometry analysis[2,3]. This approach avoids the potential disulfide scramble issue. The purified D'D3 monomer was firstly mixed with 20mM NEM in 10mM Tris-HCl pH7.4, 100mM NaCl at room temperature for 1h to block all the free cysteines. The excess NEM was removed by repeated buffer exchange. The NEM-modified D'D3 monomers were then treated with 8M Urea and 10mM DTT in 50mM Tris-HCl pH8.0, 100mM NaCl at room temperature for 1 hour to break all the intramolecular disulfide bonds. Subsequently, all the reduced cysteines were alkylated with 45mM 4-VP. All the samples were desalted and treated with protease trypsin or Asp-N.

All mass spectrometric experiments for both D'D3 dimers lysed by CNBr and D'D3 monomers cleaved by trypsin or Asp-N were performed on a Orbitrap LUMOS mass spectrometer connected to an Easy-nLC 1200 via an Easy Spray (Thermo Fisher Scientific). The peptides mixtures were loaded onto a 20cm with 0.075mm inner diameter column packed with C18 1.7-µm Reversed Phase resin (BEH, waters), and

eluted within a 60 minutes linear gradient from 95% solvent A (0.1% formic acid / 2% acetonitrile / 98% water) to 35% solvent B (0.1% formic acid / 100% acetonitrile) at a flow rate of 300nl/min. The spray voltage was set to 2KV and the temperature of ion transfer capillary was set at 275°C. One full MS scan from 350 to 1500 m/z was acquired at high resolution =120,000 (defined at m/z=400), and then about thirty most abundant multiply charged ions (including 1 charged peptide) by filter dynamic exclusion within 2-second cycle-time were followed by HCD fragmentation at resolution =30,000 (defined at m/z=400).

To identify peptide linked by Cys-Cys bond, the MS/MS ion spectra of D'D3 dimer lysed by CNBr were analyzed using pLink 2.3.9[4]. The DDA raw data were searched against the fasta database of each mutated VWF protein. pLink search parameters are as follows: precursor mass tolerance 10 p.p.m., fragment mass tolerance 20 p.p.m., cross-linker composition type including disulfide bond (HCD-SS), variable modification Oxidation (M), Homoserine (M), and Homoserine-lactone (M); peptide length minimum 4 amino acids and maximum 60 amino acids per chain, peptide mass minimum 400 and maximum 6,000 Da per chain; allowing 1 missed cleavage sites per chain. The result filter tolerance was set at 10 p.p.m., and Separate FDR was set at < 1% of the spectral level.

To identify the Cys modifications, the MS/MS ion spectra of D'D3 monomer modified by NEM or 4-VP were analyzed using SepctroMine. The DDA raw data were searched against the VWF protein fasta database. The default BGS factory setting was used except that the digestion enzyme was allowed for 1 missed specialized cleavage, with minimum peptide length set to 4, no fixed modification but with variable modification for Oxidation (M), 4-VP (C) and NEM (C), and False discovery rate (FDR) set to 1% for protein and peptide spectrum matches.

Cryo-EM sample preparation and data acquisition

The purified VWF D'D3-wt dimer or D'D3-R1136M-E1143M dimer complexed with D1D2 dimer were concentrated and incubated at 37 °C for 4 hours in MES buffer (20mM MES pH6.0, 100mM NaCl, 10mM $CaCl_2$). Aliquots (4 μL) of the protein sample were placed on glow-discharged holey carbon grids (Quantifoil Cu R1.2/1.3 +

2 nm Carbon, 300 mesh, or Quantifoil Au R1.2/1.3, 300 mesh). The grids were blotted and flash-frozen in liquid ethane cooled by liquid nitrogen with Vitrobot (Mark IV, Thermo Fisher Scientific).

The grids sample quality was verified with an FEI Talos Arctica (Thermo Fisher Scientific) 200-kV electron microscope. The verified grids with optimal ice thickness and particle density were transferred to a Titan Krios operating at 300 kV and equipped with Gatan K3 Summit detector (Gatan Inc.). Micrographs were recorded in the super-resolution mode with a calibrated pixel size of 0.485A°. Each movie has a total accumulated exposure of 50 e−/Å2 fractionated in 32 frames exposure. The final image was binned 2-fold to a pixel size of 0.97 Å. SerialEM [5] was used for the fully automated data collection. The defocus value was set from -1.0 to -2.0 μm. Data collection statistics are summarized in Supplementary Table 1. In brief, Motion Correction (MotionCo2) [6], CTF-estimation (GCTF) [7] were automatically executed by TsinghuaTitan.py program (developed by Dr. Fan Yang) during data collection.

Cryo-EM data processing

Initially, particles were auto-picked by Gautomatch (http://www.mrc-lmb.cam.ac.uk/kzhang/) and RELION 3.0 [8] from 2307 micrographs. All subsequent 2D and 3D classifications and auto-refinement were performed using RELION 3.0 or RELION 3.1[9]. For VWF D’D3-R1136M-E1143M dimer complexed with D1D2 dimer, 557,256 particles were selected after several rounds 2D and 3D classification and applied for 3D auto-refine, resulting in a helical tubule structure map at 4.4Å resolution but with relatively poor map density. A mask on 2 repeating units of filament map was applied to improve the map density of VWF D1D2-D’D3 tubule repeating units. Finally, the 2 units map containing 466,373 particles yielded a final resolution at 3.29 Å (FSC=0.143) after both CTF refinement and Bayesian Polishing were applied in RELION 3.1. For VWF D’D3-wt dimer complexed with D1D2 dimer, 1,609,923 particles were selected after 2D classification and applied for 3D auto-refine, resulting in a similar helical tubule structure map at 3.88Å resolution, followed by focused 3D classification with a mask applied on 1 repeating unit. Particles belong to the best class were subjected to removal of duplicates, resulting in 154,669 particles. Further focused

Auto-refinement was applied, which yielded a reconstruction at 3.40 Å resolution (FSC=0.143). Data processing statistics are summarized in Supplementary Table 1.

Model building and structure refinement

The D'D3 monomer models (PDB 6N29) and the D1D2 structures of mucin 2 (PDB 6TM2) were fitted into EM density map and further manually adjusted with COOT [10]. The atomic models of the quadruple complex were refinement using Phenix [11] in real space with secondary structure and geometry restraints. The final structures were validated using Molprobity web application [12]. UCSF Chimera [13] and PyMol [14] were used for map segmentation and figure generation. Model refinement statistics are summarized in Supplementary Table 1. The cryo-EM density maps and atomic coordinates have been deposited in Electron Microscopy Data Bank and Protein Data Bank with accession codes EMD-32621 and PDB 7WN4 for D'D3-wt dimer complexed with D1D2, and EMD-32622 and PDB 7WN6 for D'D3-R1136M-E1143M dimer complexed with D1D2 respectively.

References:

1 L'Abbe, D., Bisson, L., Gervais, C., Grazzini, E. & Durocher, Y. Transient Gene Expression in Suspension HEK293-EBNA1 Cells. *Methods Mol Biol* **1850**, 1-16, doi:10.1007/978-1-4939-8730-6_1 (2018).

2 Zhou, A. Functional structure of the somatomedin B domain of vitronectin. *Protein Sci* **16**, 1502-1508, doi:10.1110/ps.072819107 (2007).

3 Andreev, Y. A., Kozlov, S. A., Vassilevski, A. A. & Grishin, E. V. Cyanogen bromide cleavage of proteins in salt and buffer solutions. *Anal Biochem* **407**, 144-146, doi:10.1016/j.ab.2010.07.023 (2010).

4 Chen, Z. L. *et al.* A high-speed search engine pLink 2 with systematic evaluation for proteome-scale identification of cross-linked peptides. *Nat Commun* **10**, 3404, doi:10.1038/s41467-019-11337-z (2019).

5 Mastronarde, D. N. Automated electron microscope tomography using robust prediction of specimen movements. *J Struct Biol* **152**, 36-51, doi:10.1016/j.jsb.2005.07.007 (2005).

6 Zheng, S. Q. *et al.* MotionCor2: anisotropic correction of beam-induced motion for improved cryo-electron microscopy. *Nat Methods* **14**, 331-332, doi:10.1038/nmeth.4193 (2017).

7 Zhang, K. Gctf: Real-time CTF determination and correction. *J Struct Biol* **193**, 1-12, doi:10.1016/j.jsb.2015.11.003 (2016).

8 Zivanov, J. *et al.* New tools for automated high-resolution cryo-EM structure

determination in RELION-3. *Elife* **7**, doi:10.7554/eLife.42166 (2018).
9 Zivanov, J., Nakane, T. & Scheres, S. H. W. Estimation of high-order aberrations and anisotropic magnification from cryo-EM data sets in RELION-3.1. *IUCrJ* **7**, 253-267, doi:10.1107/S2052252520000081 (2020).
10 Emsley, P., Lohkamp, B., Scott, W. G. & Cowtan, K. Features and development of Coot. *Acta Crystallogr D Biol Crystallogr* **66**, 486-501, doi:10.1107/S0907444910007493 (2010).
11 Afonine, P. V. *et al.* Real-space refinement in PHENIX for cryo-EM and crystallography. *Acta Crystallogr D Struct Biol* **74**, 531-544, doi:10.1107/S2059798318006551 (2018).
12 Williams, C. J. *et al.* MolProbity: More and better reference data for improved all-atom structure validation. *Protein Sci* **27**, 293-315, doi:10.1002/pro.3330 (2018).
13 Pettersen, E. F. *et al.* UCSF Chimera--a visualization system for exploratory research and analysis. *J Comput Chem* **25**, 1605-1612, doi:10.1002/jcc.20084 (2004).
14 DeLano, W. L. The PyMOL Molecular Graphics System. *on World Wide Web* http://www.pymol.org (2002).
